# Supplementary material for: Isolation and Characterization of a Novel Vibrio natriegens—Infecting Phage and Its Potential Therapeutic Application in Abalone Aquaculture
Source: Biology (Basel). 2022 Nov 17;11(11):1670. doi: 10.3390/biology11111670 (PMC9687132; doi:10.3390/biology11111670)
Supplement: Supplementary file 1 [file biology-11-01670-s001.zip › Supplementary Figures.pdf]

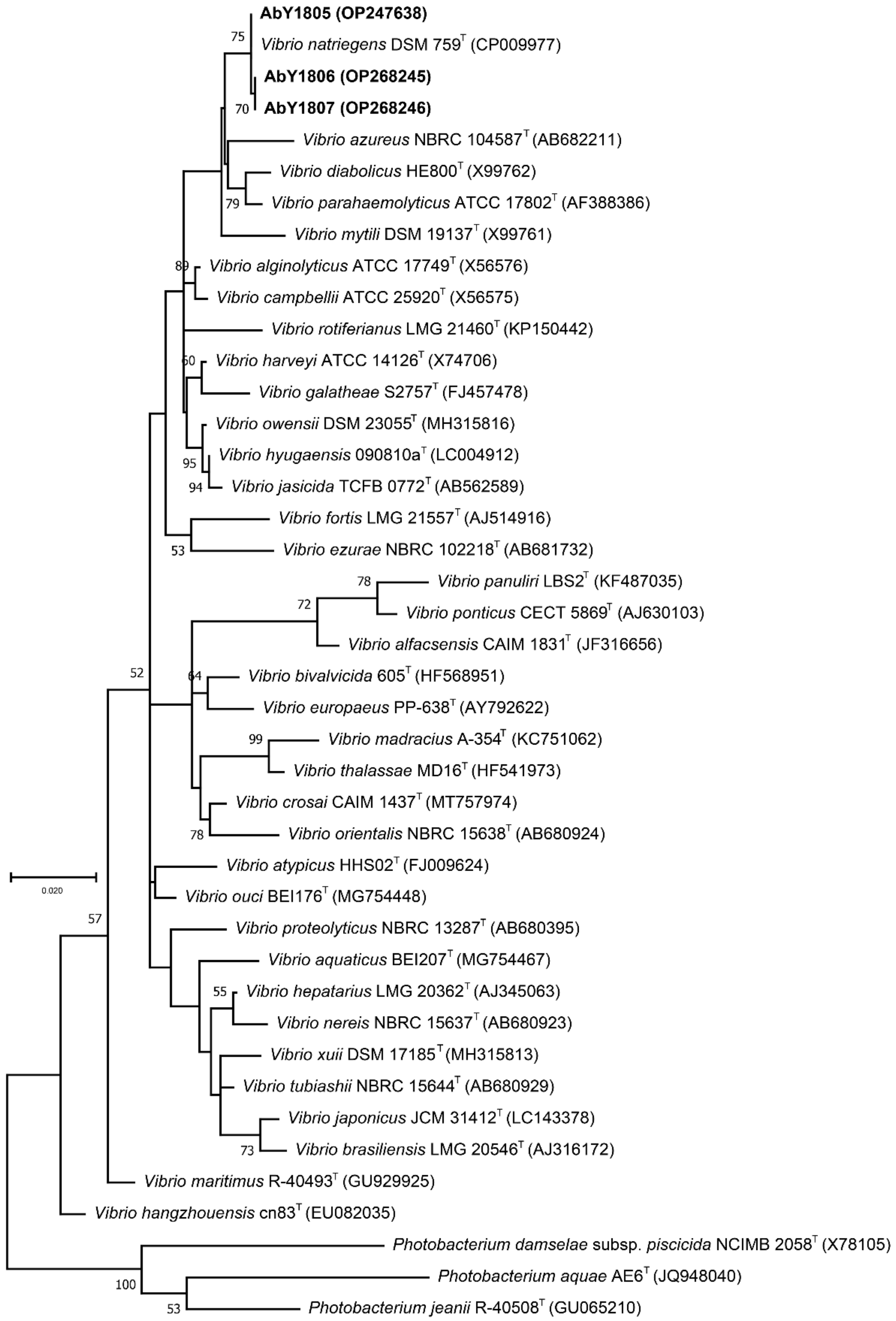

Figure S1. Maximum likelihood phylogenetic tree based on bacterial 16S rRNA gene sequences. Bootstrap values (1000 resampling) higher than 50% are shown near the corresponding nodes. The 16S rRNA gene sequences of the three pathogenic strains obtained in this study are shown in bold.

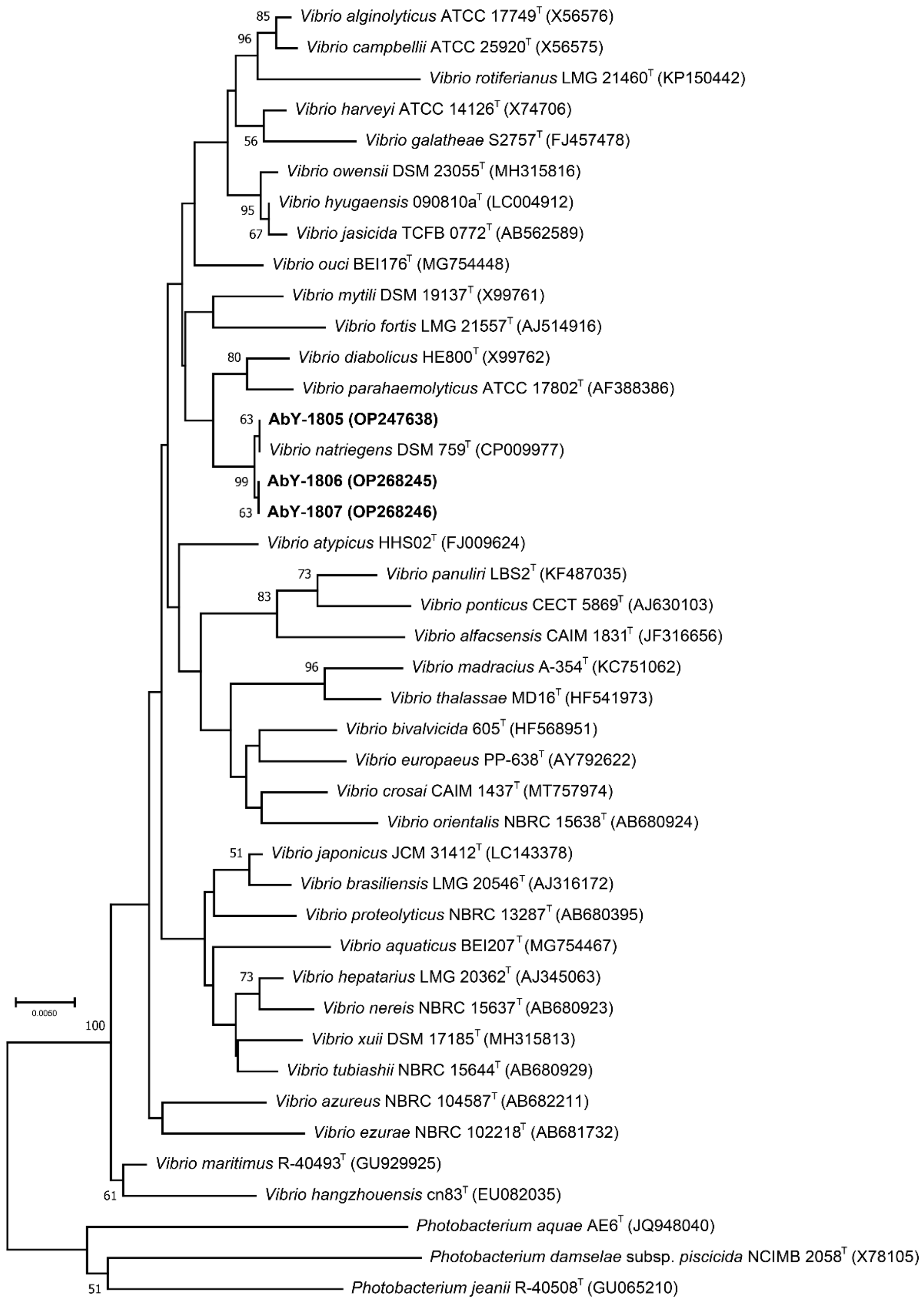

Figure S2. Minimum evolution phylogenetic tree based on bacterial 16S rRNA gene sequences. Bootstrap values (1000 resampling) higher than 50% are shown near the corresponding nodes. The 16S rRNA gene sequences of the three pathogenic strains obtained in this study are shown in bold.
