# Supplementary material for: Isolation and Characterization of a Novel Vibrio natriegens—Infecting Phage and Its Potential Therapeutic Application in Abalone Aquaculture
Source: Biology (Basel). 2022 Nov 17;11(11):1670. doi: 10.3390/biology11111670 (PMC9687132; doi:10.3390/biology11111670)
Supplement: Supplementary file 1 [file biology-11-01670-s001.zip › Supplementary Tables.pdf]

Table S1. Host range of phage vB_VnaS-L3.

Strains	Best matched species (% Identity of 16S rDNA)	GenBank ID	Source and location	Susceptibility to phage vB_VnaS-L3 1
AbY-1805	*Vibrio natriegens* (100)	OP247638	Cells of *Haliotis discus hannai* Ino, China	+
AbY-1806	*Vibrio natriegens* (99.93)	OP268245	Cells of *Haliotis discus hannai* Ino, China	+
AbY-1807	*Vibrio natriegens* (99.86)	OP268246	Larval rearing water, China	+
MCCC 1A14388	*Vibrio natriegens* (99.93)		Shrimp farm water, China	-
MCCC 1K03861	*Vibrio natriegens* (99.93)		Sediment of oyster farming area, China	-
MCCC 1H000251	*Vibrio natriegens* (100)		Type strain	-
ScY-2105	*Vibrio alginolyticus* (99.72)	OP268247	Cells of *Mizuhopecten yessoensis*, China	-
ScY-2106	*Vibrio alginolyticus* (99.93)	OP268248	Cells of *Azumapecten farreri*, China	-
ScY-2108	*Vibrio alginolyticus* (99.93	OP268249	Cells of *Azumapecten farreri*, China	-
WaY-2220	*Vibrio alginolyticus* (99.72)	OP268250	Larval rearing water, China	-
ScY-2203	*Vibrio splendidus* (99.93)	OP268251	Cells of *Azumapecten farreri*, China	-
ScY-2204	*Vibrio splendidus* (99.93)	OP268252	Cells of *Azumapecten farreri*, China	-
ScG-2206	*Vibrio splendidus* (100.00)	OP268253	Cells of *Azumapecten farreri*, China	-
WaY-2207	*Vibrio splendidus* (100.00)	OP268254	Coastal seawater, China	-

ScY-2109	*Vibrio atlanticus* (99.93)	OP268255	Cells of *Azumapecten farreri*, China	-
ScG-2110	*Vibrio atlanticus* (99.92)	OP268256	Cells of *Azumapecten farreri*, China	-
AbG-1906	*Vibrio chagasii* (100.00)	OP268257	Cells of *Haliotis discus hannai* Ino, China	-
AbG-1907	*Vibrio chagasii* (100.00)	OP268258	Cells of *Haliotis discus hannai* Ino, China	-
WaY-2101	*Vibrio chagasii* (99.51)	OP268259	Larval rearing water, China	-
AbY-1902	*Vibrio mediterranei* (99.86)	OP268260	Cells of *Haliotis discus hannai* Ino, China	-
AbY-1905	*Vibrio mediterranei* (100.00)	OP268261	Cells of *Haliotis discus hannai* Ino, China	-
AbY-2104	*Vibrio mediterranei* (100.00)	OP268262	Cells of *Haliotis discus hannai* Ino, China	-
ScG-2205	*Vibrio crassostreae* (99.78)	OP268263	Cells of *Azumapecten farreri*, China	-
AbY-2219	*Vibrio crassostreae* (99.79)	OP268264	Cells of *Haliotis discus hannai* Ino, China	-
AbY-1901	*Vibrio owensii* (100.00)	OP268265	Cells of *Haliotis discus hannai* Ino, China	-
WaY-2217	*Vibrio parahaemolyticus* (99.79)	OP268266	Coastal seawater, China	-
AbY-1904	*Vibrio neocaledonicus* (100.00)	OP268267	Cells of *Haliotis discus hannai* Ino, China	-
ScG-2111	*Vibrio celticus* (100.00)	OP268268	Cells of *Azumapecten farreri*, China	-
WaG-2102	*Shewanella japonica* (99.93)	OP268269	Larval rearing water, China	-
AbG-2103	*Shewanella japonica* (100.00)	OP268270	Cells of *Haliotis discus hannai* Ino, China	-
ScG-2112	*Shewanella japonica* (99.93)	OP268271	Cells of *Azumapecten farreri*, China	-

AbG-1908	*Shewanella waksmanii* (99.93)	OP268272	Cells of *Haliotis discus hannai* Ino, China	-
ScG-2113	*Shewanella colwelliana* (100.00)	OP268273	Cells of *Azumapecten farreri*, China	-
AbY-1903	*Photobacterium jeanii* (99.78)	OP268274	Cells of *Haliotis discus hannai* Ino, China	-
ScY-2107	*Photobacterium rosenbergii* (99.84)	OP268275	Cells of *Azumapecten farreri*, China	-
Wa-1801	*Idiomarina* sp. (99.93)	OP268276	Surface seawater, Yellow sea, China	-
Wa-1802	*Pseudoalteromonas* sp. (100.00)	OP268277	Surface seawater, Yellow sea, China	-
Wa-1803	*Pseudoalteromonas marina* (99.93)	OP268278	Surface seawater, Yellow sea, China	-
Wa-1804	*Sulfitobacter* sp. (99.97)	OP268279	Larval rearing water, China	-

1. +, susceptible; -, resistant.

Table S2. Antimicrobial resistance and susceptibility of *V. natriegens* AbY-1805

Antibiotic class/group	Antibiotic name	Concentration (μg disc^{-1})	Standard of inhibition zone（mm）			Diameter of inhibition zone	Interpretive category result
			Resistant	Intermediate	Sensitive		
Penicillin	Penicillin G	10 U	≤ 11	12~15	≥ 16	15	I
β-Lactam/β-Lactamase Inhibitor	Oxacillin	1	≤ 10	11~12	≥ 13	No zone	R
	Ampicillin	10	≤ 13	14~16	≥ 17	17	S
	Carbenicillin	100	≤ 19	20~22	≥ 23	14	R
	Piperacillin	100	≤ 17	18~20	≥ 21	21	S
Cephalosporin/Cephem	Cefalexin	30	≤ 14	15~17	≥ 18	13	R
	Cefazolin	30	≤ 14	15~17	≥ 18	12	R
	Cefradine	30	≤ 14	15~17	≥ 18	12	R

	Cefuroxime	30	≤ 14	15~22	≥ 23	14	R
	Ceftazidime	30	≤ 14	15~17	≥ 18	17	I
	Ceftriaxone	30	≤ 13	14~20	≥ 21	20	I
	Cefoperazone	75	≤ 15	16~20	≥ 21	19	I
Aminoglycosides	Amikacin	30	≤ 14	15~16	≥ 17	9	R
	Gentamicin	10	≤ 12	13~14	≥ 15	10	R
	Kanamycin	30	≤ 13	14~17	≥ 18	9	R
	Neomycin	30	≤ 12	13~16	≥ 17	10	R
Tetracycline	Tetracycline	30	≤ 14	15~18	≥ 19	No zone	R
	Oxytetracycline	30	≤ 14	12~18	≥ 19	21	S
	Minocycline	30	≤ 14	15~18	≥ 19	No zone	R
	Doxycycline	30	≤ 12	13~15	≥ 16	No zone	R
Macrolides	Erythrocin	15	≤ 13	14~22	≥ 23	10	R
	Medemycin	30	≤ 13	14~17	≥ 18	11	R
Fluoroquinolones	Norfloxacin	10	≤ 12	13~16	≥ 17	19	S
	Ofloxacin	5	≤ 12	13~15	≥ 16	19	S
	Ciprofloxacin	5	≤ 15	16~20	≥ 21	22	S
Glycopeptides	Vancomycin	30	≤ 14	15~16	≥ 17	No zone	R
polymyxins	Polymyxin B	300 IU	≤ 8	9~11	≥ 12	13	S
Folate Pathway Inhibitor	Sulfamethoxazole-trimethoprim	23.75/1.25	≤ 12	13~15	≥ 16	22	S
Nitrofuran	Furazolidone	300	≤14	15~16	≥ 17	12	S
Phenicols	Chloramphenicol	30	≤12	13~17	≥ 18	24	S
Lincosamide	Clindamycin	2	≤14	15~20	≥ 21	No zone	R
Florfenicol	Florfenicol	30	≤ 12	13~17	≥ 18	17	I

Note: S = susceptible; I = intermediate; R = resistant. The susceptibility of the strain AbY-1805 to antibiotics (Hangwei, Hangzhou, China, except for oxytetracycline and florfenicol)

was tested by using disk-diffusion method followed Kirby-Bauer disk diffusion method and the standard techniques of the Clinical and Laborarory Standard Institute (CLSI) [102,103]. Briefly, the disks were dispensed on the surface of the RO agar medium that was mixed with the strain AbY-1805, and incubated at 28 °C for 24 h. Diameters of the inhibition zone were interpreted according to the Hangwei antibiotic susceptible zone interpretation chart and the M45 guideline for Vibrio spp. (not V. cholerae) of CLSI [103], except for oxytetracycline that was interpreted according to the M2-A8 guideline of CLSI [104].

Table S3. Genome annotation of *V. natriegens* AbY-1805-infecting phage vB_VnaS-L3

ORF	Start	Stop	Strand	Putative function	CD accession no.	Best matched species	Identity (%)
1	46	528	+	Hypothetical protein	AAW67525.1	Listonella phage φHSIC	96.9
2	540	944	+	Hypothetical protein	AAW67526.1	Listonella phage φHSIC	92.5
3	1034	4273	+	Putative tail tape measure protein	AAW67528.1	Listonella phage φHSIC	77.9
4	4273	5859	+	Hypothetical protein	AAW67529.1	Listonella phage φHSIC	83.9
5	5913	8096	+	Hypothetical protein	AUG88486.1	Vibrio phage Vp-R1	47.7
7	8313	8627	+	Hypothetical protein	AGN34328.1	Vibrio phage pYD38-B	96.8
8	8727	9059	-	Hypothetical protein	AUR86940.1	Vibrio phage 1.091.O.-10N.286.52.B12	81.5
9	9196	9402	-	Hypothetical protein	AGN34330.1	Vibrio phage pYD38-B	91.2
11	9610	9816	-	Hypothetical protein	AUR92455.1	Vibrio phage 1.172.O.-10N.261.52.F5	69.1
12	10180	10389	-	Hypothetical protein	AGN34331.1	Vibrio phage pYD38-B	73.9
13	10386	10538	-	Hypothetical protein	AGN34332.1	Vibrio phage pYD38-B	86
14	10602	11060	-	Putative phage lysozyme	AAW67534.1	Listonella phage φHSIC	90.1
15	11057	11236	-	Hypothetical protein	AVR75884.1	Vibrio phage ValSw3-3	97
17	11328	11876	-	hypothetical protein	AGN34334.1	Vibrio phage pYD38-B	77.5
18	11873	12052	-	hypothetical protein	AGN34335.1	Vibrio phage pYD38-B	94.9
19	12049	12255	-	Hypothetical protein	AGN34336.1	Vibrio phage pYD38-B	89.7
20	12248	12547	-	Hypothetical protein	ADM26694.1	Pseudoalteromonas phage H105/1	53.5
21	12540	12725	-	Hypothetical protein	AZF88631.1	Vibrio phage P23	95.1

22	12750	12965	+	Hypothetical protein	AAW67537.1	Listonella phage φHSIC	69
23	12991	13374	-	hypothetical protein	AAW67538.1	Listonella phage φHSIC	96.8
24	13371	15230	-	Putative helicase	AAW67539.1	Listonella phage φHSIC	83.4
25	15199	15849	-	Hypothetical protein	AIM40751.1	Idiomarinaceae phage 1N2-2	34.9
26	15849	16403	-	Hypothetical protein	AAW67540.1	Listonella phage φHSIC	95.6
27	16388	17242	-	Hypothetical protein	AGH16091.1	Vibrio phage pYD21-A	62.3
28	17280	17813	-	Hypothetical protein	AAW67542.1	Listonella phage φHSIC	93.8
29	17820	18530	-	Putative helicase subunit	AAW67543.1	Listonella phage φHSIC	96.2
31	18922	21234	-	Putative DNA primase/polymerase	QXO10371.1	Proteus phage vB_PmiM_ZX7	41.4
32	21711	22262	+	Putative DNA polymerase III beta subunit	AAW67499.1	Listonella phage φHSIC	92.9
33	22265	22405	+	Hypothetical protein	AVR75863.1	Vibrio phage ValSw3-3	80.4
34	22544	23059	+	Coil containing protein	QGZ13264.1	Vibrio phage NF	64.6
35	23139	23312	+	Protein of unknown function DUF551	AUR89397.1	Vibrio phage 1.122.B.-10N.286.46.F8	83.6
36	23309	23461	+	Hypothetical protein	AZF88614.1	Vibrio phage P23	67.4
37	23458	23760	+	Hypothetical protein	AVR75857.1	Vibrio phage ValSw3-3	81.8
38	23790	24635	+	DNA-binding domain protein	AUR99418.1	Vibrio phage 1.265.O.-10N.286.52.F6	52.9
39	24632	24844	+	Hypothetical protein	AGN34354.1	Vibrio phage pYD38-B	87.1
40	24841	25125	+	Hypothetical protein	AZF88608.1	Vibrio phage P23	93.6
41	25184	25648	+	DNA-packaging protein	AZF88606.1	Vibrio phage P23	98.7
42	25635	27233	+	Terminase large subunit	AZF88605.1	Vibrio phage P23	97.6
43	27353	27805	+	Hypothetical protein	AGH16106.1	Vibrio phage pYD21-A	83.3
44	28012	28464	+	Hypothetical protein	AAW67508.1	Listonella phage φHSIC	88.9
45	28529	29023	+	Hypothetical protein	AVR75852.1	Vibrio phage ValSw3-3	45.6

46	29079	30509	+	Hypothetical protein	AGN34361.1	Vibrio phage pYD38-B	95
48	31096	32166	+	Putative NAD-asparagine ribosyltransferase	AAW67511.1	Listonella phage φHSIC	94.5
49	32264	32578	+	Hypothetical protein	AGN34363.1	Vibrio phage pYD38-B	84
50	32575	32763	+	Hypothetical protein	AGN34365.1	Vibrio phage pYD38-B	82.3
52	33459	34208	+	DNA methylase, partial	AUR81467.1	Vibrio phage 1.007.O.-10N.261.55.F9	68.5
53	34302	34976	+	Hypothetical protein	AGN34367.1	Vibrio phage pYD38-B	95.5
54	34987	35934	+	Major capsid protein	AVR75838.1	Vibrio phage ValSw3-3	97.4
55	35965	36555	+	Hypothetical protein	AAW67516.1	Listonella phage φHSIC	83.8
56	36861	37358	+	Hypothetical protein	AGN34370.1	Vibrio phage pYD38-B	92.1
57	37362	38159	+	Putative DNA methylase	AIM40744.1	Idiomarinaceae phage 1N2-2	67.2
58	38159	38560	+	Head-closure protein	AZF88585.1	Vibrio phage P23	92.4
59	38565	39035	+	Hypothetical protein	AVR75833.1	Vibrio phage ValSw3-3	95.5
60	39035	39418	+	Hypothetical protein	AAW67523.1	Listonella phage φHSIC	91.3
61	39489	39965	+	Hypothetical protein	AAW67524.1	Listonella phage φHSIC	98.7
